# Pediatric percentiles for transient elastography measurements - effects of age, sex, weight status and pubertal stage

**DOI:** 10.3389/fendo.2022.1030809

**Published:** 2022-09-27

**Authors:** Lina Brunnert, Ika Damayanti Puasa, Antje Garten, Melanie Penke, Susanne Gaul, Nico Grafe, Thomas Karlas, Wieland Kiess, Gunter Flemming, Mandy Vogel

**Affiliations:** ^1^ Center for Pediatric Research, University Hospital for Children and Adolescents, Leipzig, Germany; ^2^ Clinic and Polyclinic for Cardiology, Leipzig University Medical Center, Leipzig, Germany; ^3^ Leipzig Research Center for Civilization Diseases, LIFE Child, Leipzig, Germany; ^4^ Department of Medicine II, Division of Gastroenterology, Leipzig University Medical Center, Leipzig, Germany

**Keywords:** non-alcoholic fatty liver disease – NAFLD, fibroscan, liver stiffness, reference values, obesity, pediatrics

## Abstract

**Background and aims:**

Transient Elastography is a non-invasive, cost-efficient, non-ionizing, observer-independent and reliable method to detect liver fibrosis using Liver Stiffness Measurement (LSM) and the degree of fat accumulation in the liver using Controlled Attenuation Parameter (CAP). This study aims to derive reference values for both measures from healthy children and adolescents. Further, we aim to assess the potential influence of age, sex, puberty, and BMI-SDS on CAP and LSM.

**Methods:**

Within the LIFE Child study, amongst others, anthropometric data and pubertal status were assessed. Transient Elastography (TE) was performed using the FibroScan^®^ device in a population-based cohort at 982 study visits of 482 healthy children aged between 10 and 18 years. Percentiles for LSM and CAP were estimated, and the effects of age, sex, puberty and weight status were assessed through hierarchical regression models.

**Results:**

There was a strong age dependency for LSM with higher values for older children, most pronounced in the upper percentiles in boys. Contrarily, CAP was relatively stable across the age span without considerable difference between boys and girls. We found a significant positive correlation between BMI-SDS and both CAP and LSM for BMI-SDS >1.28. For BMI-SDS < 1.28, the association was also positive but reached statistical significance only for CAP. Further, the association between BMI-SDS and CAP was significantly stronger in younger than in older children. There was no association between pubertal status and CAP. For LSM, we found that children with a high BMI-SDS but not children with normal weight had significantly higher LSM values in Tanner stage 4.

**Conclusions:**

Age, sex, pubertal status and weight status should be considered when interpreting LSM and CAP in pediatric patients to facilitate and improve early detection of abnormal liver function, which is associated with common pathologies, such as NAFLD.

## Introduction

Non-alcoholic fatty liver disease (NAFLD) is the most common liver disease in children and adolescents. A recent systematic review and meta-analysis estimated a global NAFLD prevalence of 7.6% in children. Moreover, in studies focusing on children with obesity, the prevalence was as high as 34.2% (1). Due to its association with obesity, NAFLD has already become a health issue of pandemic dimensions (2). Considering the growing number of children and adolescents with obesity worldwide (3), the impact of NAFLD on public health will likely increase even further.

NAFLD can lead to liver fibrosis and cirrhosis and increases the risk of developing hepatocellular carcinoma (HCC) (4, 5). Moreover, NAFLD is associated with an increased risk of cardiovascular disease, type 2 diabetes and increased mortality at adult age (2). Detected at early stages, before the liver is irreversibly damaged, NAFLD is treatable with lifestyle modifications, e. g., improved diet, increased physical activity and weight loss (6). There is a high probability of successful development of future pharmacological treatment options, since several drugs for children with NAFLD have been tested in phase 2 trials recently (2). To facilitate successful treatment, detecting NAFLD in pediatric patients accurately at an early stage is imperative.

Until today, the gold standard for diagnosing NAFLD is the histopathological examination of a liver biopsy. However, liver biopsy in children raises several ethical issues and is therefore reluctantly performed. Children often need general anesthesia, which entails a risk for the patient. Additionally, there is the risk of bleeding or mispuncture. Furthermore, since only a tiny part of the liver is examined, there is a risk of misdiagnosis due to sampling bias (7). Hence, reliable non-invasive diagnostic tools are urgently needed.

Various serum parameters and imaging procedures have been evaluated in several studies over the last years, but mostly with rather disappointing results (2, 6, 8–10). Measurement of alanine transaminase (ALT), for instance, is the most common serum parameter for screening, but physiological levels are no reliable predictor for the absence of NAFLD. Moreover, most imaging procedures bring their own disadvantages. CT detects fibrosis and steatosis reliably; however, it must not be used regularly in pediatric patients because of radiation burden. MRI, which works without radiation and is also very sensitive, is expensive and not widely available, rendering it unsuitable to be the standard procedure for detecting NAFLD. Regular ultrasound, on the other hand, is non-ionizing, inexpensive and widely available, but not reliable in detecting NAFLD (2).

Transient Elastography (TE, by FibroScan ^®^ (Echosens, Paris, France)) has drawn a high amount of academic interest since it is a cost-efficient, observer-independent and non-ionizing method to detect fibrosis and steatosis reliably (11–18). FibroScan ^®^ provides two different methods to examine the liver: liver stiffness measurement (LSM) and controlled attenuation parameter (CAP). While LSM is a parameter to estimate liver fibrosis, CAP quantifies the percentage of liver fat.

However, to use TE in pediatric practice, reliable reference values of healthy children - including the potential influence of age, sex, weight and pubertal status - are needed. By drawing from a large, longitudinal, deeply characterized cohort of healthy children, this study aims to provide percentiles for both LSM and CAP measurement. Moreover, we will examine the potential influence of sex, age, BMI and pubertal status on these two parameters. Hereby, we hope to facilitate a better interpretation of test results and, thus, to make a beneficial contribution to pediatric practice with regard to detecting and, ideally, treating NAFLD.

## Materials and methods

This article is structured according to the STROBE (Strengthening the Reporting of Observational studies in Epidemiology) Statement checklist for cohort studies (19).

### Study design

The LIFE Child study is a prospective longitudinal population-based cohort study with a life course approach to health and disease (20). As a part of LIFE, a research project conducted at the Leipzig Research Center for Civilization Diseases, LIFE Child aims to monitor healthy child development from birth to adulthood and to understand the development of non-communicable diseases such as obesity (21). The study was designed in accordance with the Declaration of Helsinki (22). The Ethics Committee of the Medical Faculty of the University of Leipzig approved the study (Reg. No. 26410-19042010), which is registered with ClinicalTrials.gov under the clinical trial number NCT02550236.

### Setting

Fully informed and written consent was obtained from all participants (from the age of twelve) and their parents. Each study visit contained age-customized interviews, medical examinations, standardized tests, questionnaires and the collection of biological samples, as well as the implementation of FibroScan^®^ measurements (20, 21).

### Participants

Children from Leipzig or neighboring municipalities in Germany were recruited *via* advertisement at different institutions, by media or by word of mouth. The children were primarily healthy, without severe disorders like malignancies, syndromal diseases or diabetes. Accordingly, the acquired test results are qualified for generating reference values. Height was measured using a stadiometer (“Prof. Keller”; Längenmesstechnik GmbH Limbach, Limbach-Oberfrohna, Germany, measurement accuracy 0.10 cm). Participants were weighed with the “Seca701” scale (seca GmbH & Co.KG, Hamburg, Germany, accurate to 50 g). BMI was calculated and transformed into standard deviation scores (SDS) according to the guidelines of the German Obesity Association (23, 24). Overweight and obesity were defined according to the same guidelines (23, 24) as 1.28 < BMI-SDS < 1.88 and BMI-SDS ≥ 1.88, respectively. Pubertal stage was assessed according to Tanner (25, 26) by specially trained and regularly instructed investigators.

### Study size

Data from 1491 visits provided by 698 individuals from the LIFE Child cohort with a complete data set (CAP, LSM, sex, age, pubertal stage, and BMI) were available. In N=249 cases, we performed double measurements.

Our exclusion criteria were:

1. Measurements from participants younger than 10 years and older than 18 years of age were excluded (N=71 visits and 41 children), due to the small number of measurements below and above that age.
2. Participants with the intake of at least 1 of 92 potentially hepatotoxic drugs (listed in **Supplementary Table 1**) at the time of measurement were excluded, N= 62 visits and 10 children.

The remaining visits N=1358 from 647 children were used for the assessment of influence factors (sex, age, BMI-SDS, pubertal status).

For the calculation of LSM and CAP percentiles, we excluded 165 participants (231 visits) with a BMI-SDS < 3^rd^ and >97^th^ percentile (BMI-SDS < -1.88 and BMI-SDS>1.88), resulting in data from 982 visits from 482 individuals.

Glucose and insulin measurements were available from 625 visits from 196 individuals.

### Transient elastography measurement

The examination was carried out after an overnight fast by specially trained and regularly re-certified examiners. The participants were asked to lie on the back, the right arm maximally abducted, and to stay immobile during the examination. Those participants who were designated for dual measurements were asked to stay in the same position after the first measurement, and the second measurement was performed by the same examiner immediately afterwards.

LSM and CAP values were measured using the FibroScan^®^ device with the M probe (25 - 65 mm measurement depth) or XL probe (35 - 75 mm measurement depth). The FibroScan^®^ device includes the Automatic Probe Selection (APS) tool, which indicates which of the two probes should be used for measurement. LSM measures the propagation of produced shear waves, and the results are displayed in kilopascals (kPa). CAP measures the attenuation of the above-mentioned shear wave propagation, producing results in decibels per meter (dB/m). The measurement was successful when 10 valid data points could be measured.

### Laboratory parameters

Blood samples were taken from the participants after an overnight fast. Serum glucose concentrations were measured by the photometric method (Roche, Basel, Switzerland). Serum insulin concentrations were measured using a quantitative electrochemiluminescence method (Roche) (27). Homeostasis model assessment for insulin resistance (HOMA-IR) was calculated as described in Matthews et al. (28).

### Statistical analyses

Descriptive statistics are given as mean and standard deviations for continuous and counts and percentages for categorical variables.

References for LSM and CAP were estimated as a continuous function of age, stratified by sex using the LMS method as implemented in the package gamlss (29). We corrected for multiple measurements per person by setting weights on the observations accordingly. Subsequently, CAP and LSM measurements were transformed to standard deviation scores applying the new references.

Associations between LSM and CAP as outcome and the assumed predictors (sex, age, BMI-SDS, and pubertal stage) were assessed using hierarchical regression analysis. To assess the effect of puberty, raw measurements of LSM and CAP were used because of the strong dependency between age and puberty; in all other models, the age- and sex-adjusted SDS were used as outcome. All models were adjusted for multiple measurements per subject by adding the subject as random effect. The nature of associations was investigated using non-parametrical generalized additive models. The association between LSM and BMI-SDS required polynomial modeling (3^rd^ degree). Otherwise, linear approximation yielded a sufficient fit. We tested for relevant interactions between predictors. Model terms were only kept if they were necessary. In addition, models were tested for variance inflation. Results were reported as (non-standardized) coefficients and the respective 95%-confidence interval.

To assess intraobserver reliability, we calculated the overall concordance correlation coefficient (OCCC) (30, 31). In addition, we report the components overall precision (OPREC) and overall accuracy (OACCU) and present the respective Bland-Altman plots. The chosen strength-of-agreement categories are orientated to those of the Pearson product-moment correlation: CCC ≥ 0.9 (“excellent”); < 0.9 and ≥ 0.7 (“good”); < 0.7 and ≥ 0.5 (“moderate”); and < 0.5 (“low”).

The mediating effect of hepatic insulin resistance was assessed by mediation analyses using HOMA-IR implemented *via* a structural equation model.

Analyses and visualization were performed using the packages gamlss (29), lme4 (32) (version 1.1.30) and ggplot2 (3.3.6) in R (version 4.2.1; R Foundation for Statistical Computing, Vienna, Austria) (33).

## Results

### Participants

We used the data of 482 (252 male, 231 female) healthy individuals, aged between 10 and 18 years with a BMI-SDS between 3^rd^ and 97^th^ percentile, who were examined between December 2013 and June 2022 in the context of the Leipzig Research Centre for Civilization Diseases (LIFE). Since LIFE Child is a longitudinal study, some participants were measured more than once over the period of 8 years, resulting in a total of 982 (624 male, 587 female) visits for the calculation of the percentiles. Dual measurements for the evaluation of FibroScan^®^ validity were performed in 249 individuals. The population characteristics for the entire study population (N=1358) are listed in **Table 1**.

**Table 1:** Baseline characteristics of the study population.

	[ALL] N = 1358	male N = 692	female N = 666	p.overall
Sex:				
male	692 (51.0%)			
female	666 (49.0%)			
Age (years)	14.0 (2.81)	13.9 (2.87)	14.1 (2.74)	0.432
Pubertal Stage:				<0.001
1	154 (15.3%)	91 (20.5%)	63 (11.2%)	
2	146 (14.5%)	77 (17.3%)	69 (12.2%)	
3	111 (11.0%)	42 (9.46%)	69 (12.2%)	
4	170 (16.8%)	73 (16.4%)	97 (17.2%)	
5	428 (42.4%)	161 (36.3%)	267 (47.3%)	
Weight status:				0.421
underweight/normal weight	885 (65.3%)	455 (65.8%)	430 (64.8%)	
overweight	129 (9.51%)	71 (10.3%)	58 (8.73%)	
obese	342 (25.2%)	166 (24.0%)	176 (26.5%)	
BMI-SDS	0.71 (1.39)	0.64 (1.33)	0.78 (1.45)	0.055

Values are given as mean and standard deviations for continuous and counts and percentages for categorical variables.

### Reproducibility/FibroScan ^®^ validity

**Figure 1:**
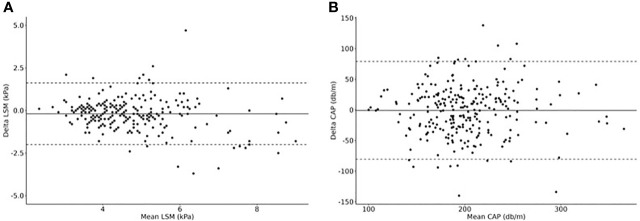
Dual measurements of **(A)** LSM and **(B)** CAP to calculate the overall concordance correlation coefficient (OCCC), the overall precision (OPREC) and the overall accuracy (OACCU). Delta values are plotted in relationship to mean values (Bland-Altman Plots). N=249 cases.

For both LSM and CAP, we could show an “excellent” OACCU. OCCC and OPREC were “good” for LSM and “moderate” for CAP. The results are shown in **Table 2** and **Figures 1A, B**.

**Table 2:** Results of the calculation of the OCCC, the OPREC and the OACCU for LSM and CAP of N=249 dual measurements.

	OCCC	OPREC	OACCU
LSM	0.74	0.76	0.97
CAP	0.66	0.66	1.0

OCCC, overall concordance correlation coefficient; OPREC, overall precision; OACCU, overall accuracy; LSM, Liver Stiffness Measurement; CAP, Controlled Attenuation Parameter.Results were classified as ≥ 0.9 “excellent”; < 0.9 and ≥ 0.7 “good”; < 0.7 and ≥ 0.5 “moderate”; and < 0.5 “low”.

### Percentiles for LSM and CAP are influenced by sex and age

**Figure 2:**
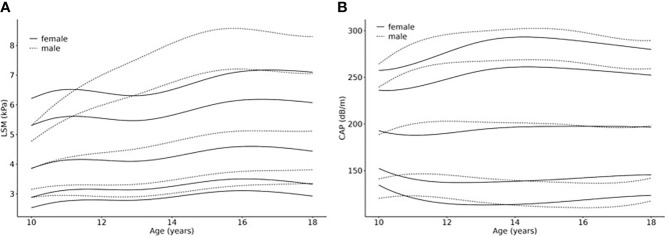
Age- and sex-related percentiles of **(A)** LSM (kPa) and **(B)** CAP (dB/m) values. Smoothed percentile curves are shown separately for females and males in relationship to age (10 – 18 years), based on a normal weight reference population from a LIFE Child study sample (N =982 cases (624 male, 587 female) of 482 (252 male, 231 female) healthy individuals). The 3^rd^ (P3), 10^th^ (P10), 50^th^ (P50, median), 90^th^ (P90) and 97^th^ (P97) percentile are shown.

The 3^rd^, 10^th^, 50^th^, 90^th^ and 97^th^ percentile curves for LSM and CAP are shown for boys and girls in **Figures 2A, B**. The respective parameter values are shown in **Supplementary Tables 2 (A)-(D)**.

LSM percentiles show increasing values for both sexes with, in general, higher values for boys, which becomes more pronounced in the upper percentiles (e.g., 16.5 years p50: girls=4.6kPa boys=5.1kPa; p97: girls=7.2kPa boys=8.5kPa). Also, the curve shapes differ from each other with regard to sex: The curves for girls ascend for the first 1.5 years, then slightly flatten for about 1.5 years, after which they ascend again until they reach their peak at about 16.5 years (P50 5.95kPa) which is followed by another slight drop in the 3^rd^, 10^th^ and 50^th^ percentile. The curves P50, P90 and P97 for boys, on the other hand, show continuous slopes until reaching their peaks, followed by a slight flattening. The age at which boys reach the highest values is comparable with that of girls (about 16.5 years) in P50, 90 and 97. In the lower percentiles, however, the highest values were measured at 18 years.

CAP percentiles show similarly shaped curves for boys and girls. The reference values are comparable as well. Comparing the reference values at the age of ten and 18 years, the lower percentiles show a tendency to descend slightly while the higher percentiles tend to ascend slightly, reaching their peaks at about 14 years. P50 depicts rather stable values during the eight years (boys: 200dB/m at age 11 and 15 years and 198db/m at age 18 years; girls: 188dB/m at age 11 years and 197dB/m from age 14.5 – 18 years).

The parameter tables are provided as part of the R package childs (version 0.8.0). The package also contains functions to transform measurement values into SDS and to create percentile curves. It is available from CRAN (https://cran.r-project.org/package=childsds).

### Influence of BMI-SDS on LSM and CAP

**Figure 3:**
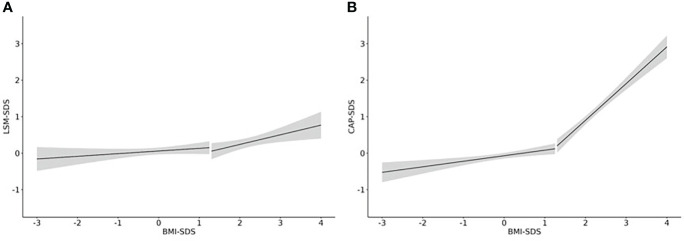
Effect of weight status on LSM and CAP. Linear regression curves including a 95%-confidence band are shown for the association of **(A)** LSM-SDS and BMI-SDS and **(B)** CAP-SDS and BMI-SDS. N = 1358 from 647 children.

After establishing percentiles, we assessed the association between weight status and both LSM and CAP values. In children with a BMI-SDS <1.28, there was a slightly positive association between LSM-SDS and BMI-SDS. However, it did not reach statistical significance (beta=0.07, p=0.48). In children with overweight and obesity, the respective effect size was three times as high, and the association became significantly positive (beta=0.26, p=0.025) (see **Figure 3A**). The two slopes were not significantly different from each other (beta_Interaction_=0.19, p=0.289). The effects of weight status on LSM were not different, regardless of age and sex.

In children with a BMI-SDS <1.28, we found a significant positive association between CAP-SDS and BMI-SDS (beta=0.15, p<0.001). In children with a BMI-SDS >1.28, the effect size was six times as high (beta=0.95, p<0.001) (see **Figure 3B**). The two slopes were significantly different from each other (beta_interaction_=0.85, p<0.001). In addition, the effect varied significantly with age, having the strongest effect for younger children (beta_10years_=1.6, p<0.001) and the weakest effect for older adolescents (beta_18years_=0.6, p<0.001). The effect of weight status on CAP did not differ between sexes.

### Influence of pubertal status on LSM

**Figure 4:**
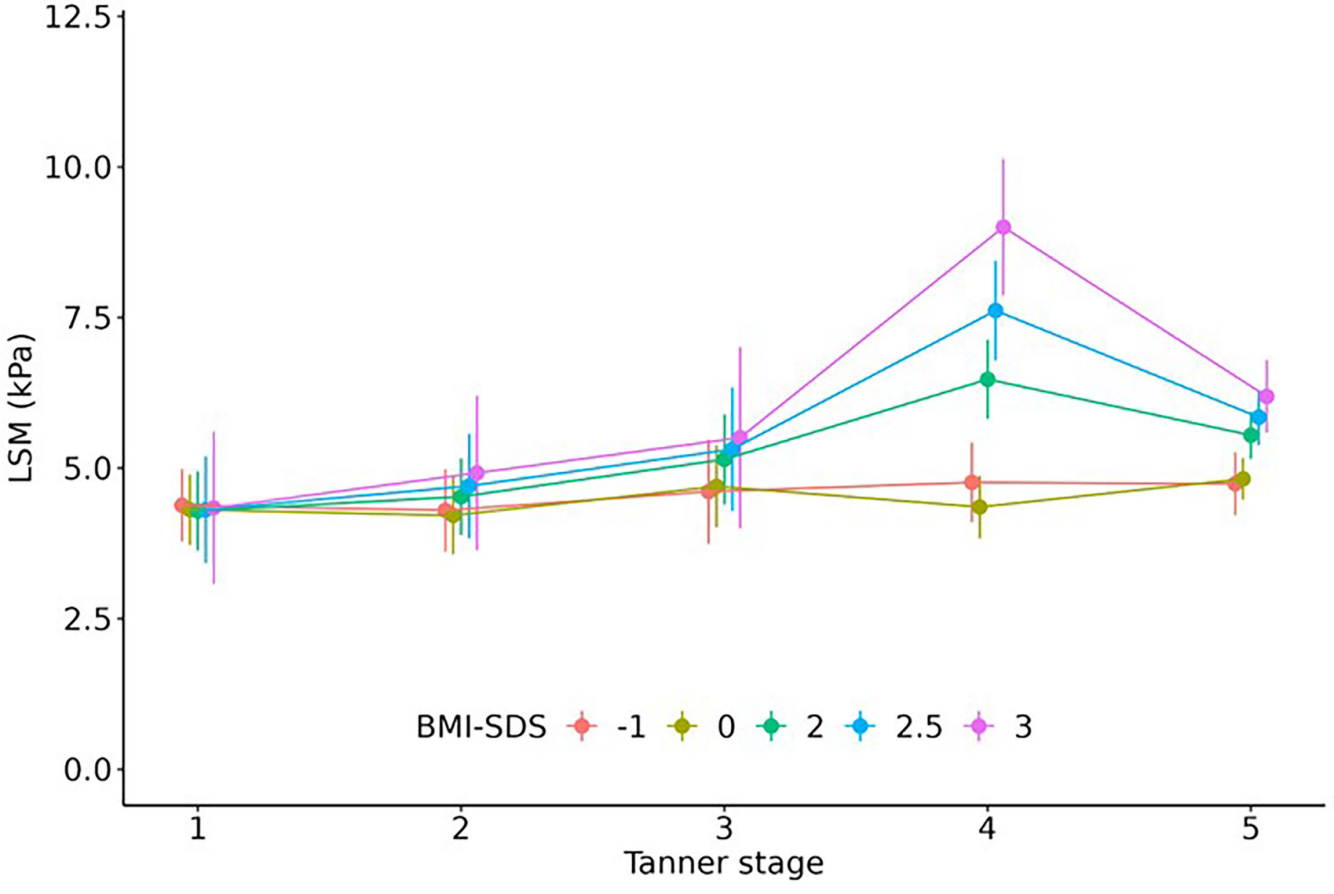
Effect of pubertal status on LSM. Children with overweight/obesity (BMI-SDS ≥ 2) but not with normal weight had significantly higher LSM values in Tanner stage 4 and 5. Regression estimates including 95% confidence interval are shown.

LSM increased significantly with advancing puberty in boys. The values were significantly higher in Tanner stage (TS) 3 (beta=1.1, p=0.029), TS 4 (beta=1.2, p=0.004), and TS 5 (beta=1.5, p<0.001) than in TS 1. In girls, there was no such distinct pattern. Considering weight status, there was a significant interaction between Tanner stage and BMI-SDS: While we found no effect of puberty in children with a BMI-SDS around or below 0, we found significantly higher LSM values for children with BMI-SDS of 1.88 or higher in TS 4 and 5. The effects were remarkably stronger in TS 4 (beta_3BMI-SDS_=4.3, p<0.001; beta_2.6BMI-SDS_=2.6, p<0.001; beta_2BMI-SDS_=1.5, p=0.005) than in TS 5 (beta_3BMI-SDS_=1.3, p=0.14; beta_2.5BMI-SDS_=1.5, p=0.004; beta_2BMI-SDS_=1.5, p=0.001) (see **Figure 4**). The association did not differ between sexes. The association of LSM with Tanner stage 4-5 was partly (approximately 1/3, p = 0.047) explained by hepatic insulin resistance, which we measured as Homeostatic Model Assessment of Insulin Resistance (HOMA-IR).

### Influence of pubertal status on CAP

There was no significant association between CAP results and puberty. Moreover, the association between BMI-SDS and CAP did not differ between Tanner stages. Values were, on average, 10 kPa higher for males than for females (p=0.012). There was no interaction between sex and Tanner stage or BMI-SDS.

## Discussion

Considering the rising prevalence of obesity and concomitant liver diseases, especially NAFLD, in children and adolescents, non-invasive diagnostic tools to accurately detect liver pathologies in pediatric patients are urgently needed. Transient Elastography has been used extensively to aid the diagnosis of fatty liver disease and fibrosis in the adult population for which TE reference values are available (34–36). Several studies have already postulated the need for reliable TE reference values for children, respectively the necessity of further detailed research on TE measurement in the pediatric context (6, 37).

With the aim to close this knowledge gap, this study provides pediatric reference values and presents the respective percentiles for the Transient Elastography measurements LSM and CAP, based on our investigation of a large and well-characterized cohort of healthy children and adolescents. We decided to include children with overweight when estimating percentiles because our analyses revealed that the influence of BMI-SDS was similar as in children with normal weight. In contrast, increasing BMI-SDS had considerably stronger effects on LSM and CAP for children with obesity (**Figure 3**).

Furthermore, we analyzed the influence of age, sex, weight and pubertal status on LSM and CAP. Thereby, we enable examiners and practitioners to interpret LSM and CAP test results in children more accurately, having appropriate reference values at hand.

Our research has shown that LSM is age-dependent, and LSM test results tend to increase with age in the pediatric context. Our reference values for LSM are generally in line with findings of other recent studies examining healthy children (38–43). Likewise, the increase over age was also observed in other studies (38–42). Contrary to this, Ramirez et al., who investigated a cohort of 462 healthy children and provided reference values as well, found no effect of age on LSM (43). Potential reasons for this inconsistency include a different age range (12 – 20 years) as well as a different ethnic and geographic background of the cohort. A recent meta-analysis with 1702 participants, on the other hand, also found that values increase with age (40). Zeng et al. (44) provided reference values for five-year-olds based on a very large cohort. The reference values they established were remarkably lower than ours. Since we included participants starting at age 10 who, from the start, showed higher values compared to those of five-year-olds (Zeng 2019: LSM_5years_ median 3.2 kPa *vs*. Brunnert 2022: LSM_10years_ median 3.9 kPa), we regard their study results, taken together with ours, as strongly supporting the validity of the assumption that pediatric LSM values increase with age. However, Mjelle et al. (39) state that there is about the same number of studies indicating an age-dependency of LSM values as there is for age-independency. This clearly highlights the need to further investigate the age-dependency of LSM values in the pediatric context. In our study, LSM values peak at 14.5 years and stay more or less stable afterwards. This leads to the assumption that after age 18 no further increase in LSM values will occur. This assumption is in line with the so far published studies of the adult population stating that LSM results show no age-dependency (45, 46).

Moreover, our research has shown that LSM test results are higher for boys. This is also confirmed by results from other relevant recent studies (38, 39, 42). Tokuhara et al., on the other hand, could not find any influence of sex on LSM results (41). Likewise, the above-mentioned study by Ramirez et al. (43) did not find sex-dependent alterations of LSM. Since our study clearly shows the sex-dependency of LSM test results, we expect that future research will further validate this outcome.

We could not identify any correlation between age, sex and CAP. With regard to pediatric CAP measurements, there are only a few published studies providing reference values. Ramirez et al. (43) presented stable, age- and sex-independent CAP values from the ages 12 to 20. Their findings are in line with our observation that CAP values are neither age- nor sex-dependent. This was also shown by a recent study by Ferraioli et al. (47). However, Zeng et al. (44) identified a median CAP value of 171db/m for five-year-olds, so there might be a tendency for lower CAP values at younger ages, if we take into account that our values for older children and adolescents are remarkably higher (median LSM at age 15: 197dB/m for girls, 200dB/m for boys). Since we only analyzed results of children aged 10 years and older, our study could not add further insights on the question of whether CAP values increase below age 10.

We found a positive correlation between weight status and LSM as well as CAP test results, also found by Zeng et al. (44). In addition, Ferraioli et al. (47) examined CAP values of children categorized as ‘normal weight’, ‘overweight’ and ‘obese’. They, too, found a significant positive association between CAP and weight status (30). Lee et al. evaluated LSM in children with obesity. Values were remarkably higher (16) than in our reference population, which further supports our finding of a considerable impact of weight status on LSM values.

Furthermore, we found that LSM but not CAP values differ across puberty. To our knowledge, we present the first examination of the impact of pubertal status on TE measurements. Partly, the effect might be explained by the increasing hepatic insulin resistance during puberty (48) as our results suggest. Another reason for increased hepatic insulin resistance is obesity (49). Accordingly, we found that adolescents with obesity had significantly higher LSM, especially in Tanner stage 4 and 5. The underlying mechanisms of this phenomenon are unclear and should be subject to future research.

Evaluating dual measurements, we could show that TE is a method with medium reproducibility. Our findings are in line with results of other studies investigating the reproducibility of TE measurements: Ferraioli et al. reported a concordance correlation coefficient (CCC) for CAP of 0.82 for children with normal weight and 0.6 for children with obesity (17). Rowland et al. reported a CCC for LSM of 0.85 (50). We would, therefore, suggest the implementation of a second measurement in case of borderline TE results, to improve the reliability of the results.

There are some limitations to our study. We only used data from a single study center with limited access to subjects with diverse ethnic background. Thus, our results are not necessarily representative of pediatric patients worldwide. Furthermore, families participating in the LIFE Child research project generally have a socio-economic status above average (51) which could also render our findings less representative with regard to both the global pediatric population as well as the general pediatric population of a particular state or region. In addition, the HOMA-IR was only available for a subpopulation (n = 196) which led to less power in the related analyses. Moreover, for evident ethical reasons, we did not perform liver biopsies to validate our test results.

Nevertheless, our study has several strengths. To our knowledge, this paper is the first to provide reference values for both LSM and CAP based on a large pediatric cohort from 10 to 18 years. Additionally, we established that age, sex, BMI-SDS and pubertal status have an impact on TE test results and, thus, should be considered when evaluating LSM and CAP values. Accordingly, we suggest our sex- and age-adapted reference values to interpret TE results in pediatric practice. There are numerous studies evaluating the usefulness and feasibility of TE for pediatric subjects, but most of them only examine patients with NAFLD or obesity. However, in pediatric practice, we need reference values guiding us in our endeavor to identify potential risks or existing diseases in patients. Thus, the reference values and percentiles we present in this paper can help us to red-flag conspicuous test results.

Given the already high and, most likely, further increasing prevalence of liver diseases such as NAFLD, it is paramount to detect potential diseases at an early stage. Our paper attempts to make a valuable contribution to this endeavor in terms of research as well as practice.

## Data availability statement

The datasets presented in this article are not readily available because data cannot be shared publicly because there exist ethical restrictions. The LIFE Child study is a study collecting potentially sensitive information. Publishing data sets is not covered by the informed consent provided by the study participants. Furthermore, the data protection concept of LIFE requests that all (external as well as internal) researchers interested in accessing data sign a project agreement. Researchers that are interested in accessing and analyzing data collected in the LIFE Child study may contact the data use and access committee (dm@life.uni-leipzig.de). Requests to access the datasets should be directed to dm@life.uni-leipzig.de.

## Ethics statement

This study was reviewed and approved by The Ethics Committee of the Medical Faculty of the University of Leipzig. Written informed consent to participate in this study was provided by the participants’ legal guardian/next of kin.

## Author contributions

Conceptualization, SG, MP, WK, AG and GF. Methodology, TK, NG, GF and MV. Software MV. Validation, IP, NG and MV. Formal analysis IP, LB and MV. Investigation, IP, NG, GF, MV. Resources, TK, GF, WK and MV. Data curation, IP, LB and MV. Writing—original draft preparation LB, IP and MV. Writing—review and editing, LB, AG, GF and MV. Visualization, IP, LB and MV. Supervision, GF, MV, AG and WK. Project administration, AG. Funding acquisition, SG, MP and WK. All authors contributed to the article and approved the submitted version.

## Funding

This publication is supported by LIFE – Leipzig Research Center for Civilization Diseases, University of Leipzig. LIFE is funded by means of the European Union, by means of the European Social Fund (ESF, https://ec.europa.eu/regional_policy/en/funding/social-fund/), by the European Regional Development Fund (ERDF, https://ec.europa.eu/regional_policy/en/funding/erdf/), and by means of the Free State of Saxony. This publication was funded by the Open Access Publishing Fund of Leipzig University supported by the German Research Foundation within the program Open Access Publication Funding. Furthermore, we acknowledge support from the Roland-Ernst-Stiftung. The funders had no role in study design, data collection and analysis, decision to publish, or preparation of the manuscript.

## Acknowledgments

We are deeply grateful to all the families who have taken part in this study, and the whole LIFE Child team.

## Conflict of interest

TK received unrestricted research grants from Echosens SA, France, not related to this project. TK took part in a clinical advisory board meeting of Echosens SA.

The remaining authors declare that the research was conducted in the absence of any commercial or financial relationships that could be construed as a potential conflict of interest.

## Publisher’s note

All claims expressed in this article are solely those of the authors and do not necessarily represent those of their affiliated organizations, or those of the publisher, the editors and the reviewers. Any product that may be evaluated in this article, or claim that may be made by its manufacturer, is not guaranteed or endorsed by the publisher.
