# Supplementary material for: Pediatric percentiles for transient elastography measurements - effects of age, sex, weight status and pubertal stage
Source: Front Endocrinol (Lausanne). 2022 Sep 27;13:1030809. doi: 10.3389/fendo.2022.1030809 (PMC9551398; doi:10.3389/fendo.2022.1030809)
Supplement: Supplementary file 1 [file Table_1.docx]

Supplementary Material

**Supplementary Table 1.** List of potentially hepatotoxic drugs. Individuals with intake of ≥ 1 of these drugs were excluded from the study population.

| Abacavir Sulfate | Flutamide | Phenazopyridine Hydrochloride |
| --- | --- | --- |
| Acetaminophen | Fluvastatin Sodium | Phenytoin Sodium |
| Allopurinol | Glimepiride | Pilocarpine Hydrochloride |
| Amiodarone Hydrochloride | Glipizide | Pravastatin Sodium |
| Amoxicillin | Glyburide | Probenecid |
| Amoxicillin Trihydrate; Clavulanate | Indinavir Sulfate | Progesterone |
| Potassium | Isoniazid | Propylthiouracil |
| Atorvastatin Calcium | Itraconazole | Pyrazinamide |
| Azathioprine | Ketoconazole | Quinine Sulfate |
| Bicalutamide | Labetalol Hydrochloride | Repaglinide |
| Bromocriptine Mesylate | Lamivudine | Rifabutin |
| Carbamazepine | Leflunomide | Ritonavir |
| Carbidopa; Levodopa | Leucovorin Calcium | Rivastigmine Tartrate |
| Cerivastatin Sodium | Lovastatin | Saquinavir |
| Chlorpropamide | Medroxyprogesterone Acetate | Simvastatin |
| Chlorzoxazone | Megestrol Acetate | Stavudine |
| Cholesterol Lowering Drug – Unspec. | Nomegestrol and Estradiol | Sulfamethoxazole |
| Clotrimazole | Mesalamine | Sulfasalazine |
| Colchicine | Mestranol; Norethindrone | Tamoxifen Citrate |
| Desogestrel; Ethinyl Estradiol | Methimazole | Terbinafine Hydrochloride |
| Diclofenac | Methotrexate Sodium | Testosterone |
| Didanosine | Methyldopa | Tetracycline Hydrochloride |
| Divalproex Sodium | Minocycline Hydrochloride | Tizanidine Hydrochloride |
| Efavirenz | Montelukast Sodium | Tolazamide |
| Erythromycin | Nabumetone | Tretinoin |
| Estrogen | Niacin | Trimethoprim |
| Estrogen and Progesteron | Nitrofurantoin | Troglitazone |
| Ethambutol Hydrochloride | Norethindrone | Valproic Acid |
| Fenofibrate | Olanzapine | Zafirlukast |
| Fluconazole | Pemoline | Zidovudine |
| Fluorouracil | Permethrin |  |

**Supplementary Table 2.** Reference values of the study reference population (N=982) for Liver Stiffness Measurement (LSM) for boys (A) and girls (B) and Controlled Attenuation Parameter (CAP) for boys (C) and girls (D) including mu, coefficient of variation (sigma) and skewness (nu), tabulated in half-year steps.

(A)

| sex | age | 3^rd^ percentile | 10^th^ percentile | 50^th^ percentile | 90^th^ percentile | 97^th^ percentile | mu | sigma | nu |
| --- | --- | --- | --- | --- | --- | --- | --- | --- | --- |
| male | 10 | 120.28 | 141.10 | 188.49 | 239.37 | 264.26 | 188.4861 | 0.2037 | 0.7283 |
| male | 10.5 | 122.45 | 144.69 | 195.44 | 250.07 | 276.83 | 195.4381 | 0.2107 | 0.7283 |
| male | 11 | 122.98 | 146.39 | 199.95 | 257.76 | 286.10 | 199.9537 | 0.2176 | 0.7283 |
| male | 11.5 | 122.11 | 146.43 | 202.21 | 262.56 | 292.18 | 202.2145 | 0.2244 | 0.7283 |
| male | 12 | 120.36 | 145.37 | 202.87 | 265.22 | 295.87 | 202.8683 | 0.2309 | 0.7283 |
| male | 12.5 | 118.18 | 143.72 | 202.56 | 266.50 | 297.97 | 202.5624 | 0.237 | 0.7283 |
| male | 13 | 116.04 | 142.00 | 201.94 | 267.21 | 299.36 | 201.9444 | 0.2424 | 0.7283 |
| male | 13.5 | 114.27 | 140.59 | 201.50 | 267.93 | 300.68 | 201.5018 | 0.2471 | 0.7283 |
| male | 14 | 112.87 | 139.50 | 201.18 | 268.55 | 301.78 | 201.1845 | 0.2508 | 0.7283 |
| male | 14.5 | 111.82 | 138.63 | 200.83 | 268.81 | 302.37 | 200.8296 | 0.2535 | 0.7283 |
| male | 15 | 111.06 | 137.92 | 200.27 | 268.46 | 302.13 | 200.2739 | 0.2549 | 0.7283 |
| male | 15.5 | 110.54 | 137.28 | 199.35 | 267.24 | 300.76 | 199.3544 | 0.2549 | 0.7283 |
| male | 16 | 110.26 | 136.69 | 198.02 | 265.04 | 298.13 | 198.0154 | 0.2534 | 0.7283 |
| male | 16.5 | 110.48 | 136.47 | 196.68 | 262.42 | 294.86 | 196.6814 | 0.2504 | 0.7283 |
| male | 17 | 111.53 | 137.00 | 195.91 | 260.12 | 291.78 | 195.9117 | 0.2457 | 0.7283 |
| male | 17.5 | 113.74 | 138.70 | 196.27 | 258.87 | 289.70 | 196.2654 | 0.2394 | 0.7283 |
| male | 18 | 117.50 | 141.99 | 198.30 | 259.37 | 289.40 | 198.3018 | 0.2314 | 0.7283 |

LSM reference values in kPa for boys, age in years.

(B)

| sex | age | 3^rd^ percentile | 10^th^ percentile | 50^th^ percentile | 90^th^ percentile | 97^th^ percentile | mu | sigma | nu |
| --- | --- | --- | --- | --- | --- | --- | --- | --- | --- |
| female | 10 | 134.54 | 152.34 | 192.77 | 236.18 | 257.44 | 192.7662 | 0.1698 | 0.6733 |
| female | 10.5 | 126.36 | 145.45 | 189.10 | 236.33 | 259.57 | 189.0982 | 0.1877 | 0.6733 |
| female | 11 | 120.70 | 141.06 | 187.94 | 239.01 | 264.23 | 187.9407 | 0.2036 | 0.6733 |
| female | 11.5 | 116.94 | 138.51 | 188.45 | 243.17 | 270.27 | 188.4518 | 0.217 | 0.6733 |
| female | 12 | 114.68 | 137.34 | 190.05 | 248.06 | 276.87 | 190.0519 | 0.2277 | 0.6733 |
| female | 12.5 | 113.56 | 137.13 | 192.16 | 252.93 | 283.15 | 192.1616 | 0.2355 | 0.6733 |
| female | 13 | 113.21 | 137.46 | 194.20 | 256.99 | 288.26 | 194.2015 | 0.2406 | 0.6733 |
| female | 13.5 | 113.31 | 137.96 | 195.69 | 259.64 | 291.51 | 195.6892 | 0.2431 | 0.6733 |
| female | 14 | 113.78 | 138.56 | 196.62 | 260.94 | 292.99 | 196.6177 | 0.2433 | 0.6733 |
| female | 14.5 | 114.59 | 139.29 | 197.13 | 261.15 | 293.05 | 197.1252 | 0.2416 | 0.6733 |
| female | 15 | 115.69 | 140.15 | 197.35 | 260.59 | 292.07 | 197.3501 | 0.2385 | 0.6733 |
| female | 15.5 | 117.03 | 141.15 | 197.43 | 259.55 | 290.44 | 197.4305 | 0.2344 | 0.6733 |
| female | 16 | 118.54 | 142.25 | 197.48 | 258.30 | 288.52 | 197.4754 | 0.2297 | 0.6733 |
| female | 16.5 | 120.09 | 143.37 | 197.46 | 256.92 | 286.43 | 197.4635 | 0.2247 | 0.6733 |
| female | 17 | 121.52 | 144.37 | 197.34 | 255.44 | 284.25 | 197.3378 | 0.2199 | 0.6733 |
| female | 17.5 | 122.69 | 145.12 | 197.04 | 253.89 | 282.04 | 197.0413 | 0.2157 | 0.6733 |
| female | 18 | 123.45 | 145.52 | 196.52 | 252.27 | 279.86 | 196.5169 | 0.2122 | 0.6733 |

LSM reference values in kPa for girls, age in years.

(C)

| sex | age | 3^rd^ percentile | 10^th^ percentile | 50^th^ percentile | 90^th^ percentile | | 97^th^ percentile | mu | sigma | nu |
| --- | --- | --- | --- | --- | --- | --- | --- | --- | --- | --- |
| male | 10 | 2.88 | 3.15 | 3.85 | 4.77 | 5.30 | | 3.8512 | 0.1617 | -0.3079 |
| male | 10.5 | 2.94 | 3.25 | 4.07 | 5.18 | 5.84 | | 4.066 | 0.1822 | -0.3079 |
| male | 11 | 2.95 | 3.29 | 4.22 | 5.52 | 6.30 | | 4.217 | 0.2011 | -0.3079 |
| male | 11.5 | 2.93 | 3.30 | 4.31 | 5.78 | 6.69 | | 4.3147 | 0.2179 | -0.3079 |
| male | 12 | 2.91 | 3.30 | 4.38 | 5.99 | 7.00 | | 4.3821 | 0.232 | -0.3079 |
| male | 12.5 | 2.90 | 3.30 | 4.44 | 6.17 | 7.27 | | 4.4424 | 0.2432 | -0.3079 |
| male | 13 | 2.91 | 3.32 | 4.52 | 6.35 | 7.53 | | 4.5185 | 0.2516 | -0.3079 |
| male | 13.5 | 2.95 | 3.38 | 4.63 | 6.55 | 7.81 | | 4.6256 | 0.2571 | -0.3079 |
| male | 14 | 3.01 | 3.46 | 4.75 | 6.75 | 8.07 | | 4.7522 | 0.2599 | -0.3079 |
| male | 14.5 | 3.09 | 3.55 | 4.88 | 6.94 | 8.30 | | 4.8812 | 0.2606 | -0.3079 |
| male | 15 | 3.17 | 3.64 | 5.00 | 7.09 | 8.48 | | 4.9956 | 0.2594 | -0.3079 |
| male | 15.5 | 3.24 | 3.71 | 5.08 | 7.19 | 8.57 | | 5.0783 | 0.2568 | -0.3079 |
| male | 16 | 3.28 | 3.76 | 5.12 | 7.20 | 8.57 | | 5.1173 | 0.2534 | -0.3079 |
| male | 16.5 | 3.31 | 3.78 | 5.12 | 7.17 | 8.50 | | 5.1231 | 0.2495 | -0.3079 |
| male | 17 | 3.32 | 3.79 | 5.11 | 7.12 | 8.41 | | 5.1129 | 0.2456 | -0.3079 |
| male | 17.5 | 3.33 | 3.79 | 5.10 | 7.07 | 8.33 | | 5.1036 | 0.2421 | -0.3079 |
| male | 18 | 3.35 | 3.81 | 5.11 | 7.05 | 8.30 | | 5.112 | 0.2394 | -0.3079 |

CAP reference values in dB/m for boys, age in years.

(D)

| sex | age | 3^rd^ percentile | 10^th^ percentile | 50^th^ percentile | 90^th^ percentile | 97^th^ percentile | mu | sigma | nu |
| --- | --- | --- | --- | --- | --- | --- | --- | --- | --- |
| female | 10 | 2.53 | 2.88 | 3.86 | 5.30 | 6.21 | 3.8611 | 0.2375 | -0.2687 |
| female | 10.5 | 2.68 | 3.04 | 4.04 | 5.51 | 6.43 | 4.0413 | 0.232 | -0.2687 |
| female | 11 | 2.76 | 3.13 | 4.14 | 5.61 | 6.52 | 4.139 | 0.2273 | -0.2687 |
| female | 11.5 | 2.79 | 3.16 | 4.16 | 5.60 | 6.50 | 4.1585 | 0.2235 | -0.2687 |
| female | 12 | 2.79 | 3.15 | 4.13 | 5.547 | 6.42 | 4.135 | 0.2204 | -0.2687 |
| female | 12.5 | 2.78 | 3.13 | 4.10 | 5.49 | 6.34 | 4.1036 | 0.218 | -0.2687 |
| female | 13 | 2.79 | 3.14 | 4.10 | 5.47 | 6.31 | 4.0994 | 0.2164 | -0.2687 |
| female | 13.5 | 2.83 | 3.18 | 4.15 | 5.53 | 6.37 | 4.1499 | 0.2153 | -0.2687 |
| female | 14 | 2.89 | 3.25 | 4.24 | 5.65 | 6.51 | 4.2437 | 0.215 | -0.2687 |
| female | 14.5 | 2.97 | 3.34 | 4.36 | 5.80 | 6.69 | 4.358 | 0.2152 | -0.2687 |
| female | 15 | 3.04 | 3.42 | 4.47 | 5.96 | 6.87 | 4.4696 | 0.2161 | -0.2687 |
| female | 15.5 | 3.09 | 3.48 | 4.56 | 6.09 | 7.03 | 4.5556 | 0.2177 | -0.2687 |
| female | 16 | 3.11 | 3.50 | 4.60 | 6.16 | 7.13 | 4.5977 | 0.2198 | -0.2687 |
| female | 16.5 | 3.09 | 3.49 | 4.60 | 6.18 | 7.17 | 4.5986 | 0.2226 | -0.2687 |
| female | 17 | 3.05 | 3.45 | 4.57 | 6.17 | 7.17 | 4.5665 | 0.226 | -0.2687 |
| female | 17.5 | 2.99 | 3.40 | 4.51 | 6.13 | 7.14 | 4.5097 | 0.2301 | -0.2687 |
| female | 18 | 2.92 | 3.32 | 4.44 | 6.07 | 7.10 | 4.4368 | 0.235 | -0.2687 |

CAP reference values in dB/m for girls, age in years.
